# Innovative health financing mechanisms: the case of Africa’s unified approach to vaccine acquisition

**DOI:** 10.1093/heapol/czad109

**Published:** 2023-11-16

**Authors:** Chiamaka P Ojiako

**Affiliations:** The Robert F. Wagner Graduate School of Public Service, New York University, 295 Lafayette St, New York, NY 10012, USA

**Keywords:** Health financing, accountability, international health

Key messages

- Long before the COVID-19 pandemic, Africa has been grappling with developing a sustainable financing model for financing vaccine procurement and rather than taking ownership the leadership outsourced their responsibility by relying on donor-driven funding models.
- During the COVID-19 pandemic, Africa took the initiative by designing and implementing innovative financing mechanisms underpinned by principles of solidarity and togetherness; ‘the whole of Africa approach’.
- Although the financing models adopted by African institutions to procure the COVID-19 vaccines were not exactly novel, the autonomy and independence they had in sourcing and managing the funds reduced cost, fragmentation and the unpredictability of external aid.
- Africa’s institution-driven approach, strategic collaborations, high political engagement and collective finance management are noteworthy for financing other vaccines and possibly addressing other health systems building blocks in Africa, including Africa’s vision of vaccine self-reliance.

Typically, discourses concerning innovative health financing mechanisms for vaccines revolve around new mechanisms that have successfully funded vaccine research, manufacturing and purchase. Meanwhile, a critical yet often overlooked aspect of innovative vaccine financing mechanisms is the challenge of striking the right balance between managing access to the finance and beneficiary autonomy. Africa’s continental response to COVID-19, which was characterized by the use of innovative vaccine procurement mechanisms, demonstrates the importance of autonomous application of external aid in promoting ownership and a more timely response to public health emergencies.

Vaccine expenditures are huge, but this commentary focuses on vaccine procurement costs, which several African countries offset through donor-led routine vaccination and co-financed vaccine production models. The continent’s inadequate capacity to meet the vaccine needs of the population has not yet been reflected in the prioritization of vaccine funding and the significant increase in vaccine investment (WHO| Regional Office for Africa, 2013). The Global Alliance for Vaccines and Immunization (GAVI) funding has played an important role in filling the vaccine funding gap in Africa, with 40 African countries out of the 57 GAVI-eligible countries receiving 65% of GAVI’s total disbursement. While GAVI’s financial support has improved access to life-saving vaccines, it has equally enabled a concerning level of complacency amongst benefiting countries with their rather slow pace of transitioning towards becoming self-financing (Gavi impact in Africa since 2000, 2022) (Cernuschi *et al.*, 2018).

The political choice to invest poorly in vaccine financing has spawned several donor-driven financing models aimed at closing Africa’s wide vaccine financing gap, from the Advanced Market Commitment adopted by GAVI and other donors to make down payments for the research and development of the pneumococcal vaccine and the Ebola vaccines (Cernuschi *et al.*, 2022) to public–private partnerships and joint ventures with funds from grants, public funds and philanthropic foundation for vaccine procurement (LaForce *et al.*, 2007). While these innovative financing mechanisms are lauded as catalytic successes, they have also been criticized for limiting the autonomy of beneficiary countries due to the strong influence of private sector financial actors (Stein, 2021; Hughes-McLure and Mawdsley, 2022). Lack of autonomy and misalignment of donor-driven vaccine financing models with national and continental priorities has stifled creativity and shifted the responsibility of national governments to development partners and global health agencies.

During the COVID-19 pandemic, the domestic financial shortage for vaccine procurement proved costly for African nations due to a monopolistic vaccine market, where greater negotiating power and financial resources became essential conditions for acquiring limited vaccines. Despite earlier pandemics’ lessons about the dangers of relying solely on imported vaccines, Africa was extremely dependent on promised equitable vaccine sharing arrangements and, as a result, was relegated to the end of the vaccine queue. But this crisis, along with broken promises of global solidarity and vaccine nationalism, sparked a concerted ‘whole of Africa’ strategy to remedy vaccine inequities. This approach led by the African Union (AU) in partnership with the Africa Centres for Disease Control and Prevention (Africa CDC), African Export-Import Bank (Afreximbank), African Development Bank (AfDB) and United Nations Economic Commission for Africa (UNECA) yielded positive results with innovations including new institutions, mechanisms and approaches for funding and managing external aid for COVID-19 vaccines (Songwe, 2022).

The $12 billion World Bank facility for COVID-19 vaccine acquisition and deployment in Africa as well as the COVAX facility doses were insufficient to meet Africa’s vaccination needs. Given the fiscal pressures Ministries of Finance were under during the pandemic, it was important to raise additional money and devise flexible mechanisms for accessing available funding. As a result, the African Vaccine Acquisition Task Team (AVATT) was formed with the mandate of supporting access and funding of the COVID-19 vaccines. Afreximbank established a $2 billion Advanced Procurement Commitment (APC) financing mechanism and a deferred Instalment Payment Plan (IPP). The APC guaranteed the contracts with vaccine manufacturers while the 5 years helped countries who couldn’t pay for their vaccine orders in cash. The APC guaranteed vaccine manufacturer contracts, while the 5-year deferred IPP helped countries that couldn’t pay for their vaccine orders in cash upon delivery (Annual Report, 2021). Countries could use the IPP regardless of their eligibility for World Bank financing facilities with the option to repay based on their fiscal position (Africa, 2021). The African Vaccine Acquisition Trust (AVAT), a pool procurement agency, was also established, and it was successful in aggregating demand, lowering costs and making better use of scarce resources. Also, to bolster vaccine confidence, the AVAT No-Fault Compensation Scheme Trust was set up to compensate people for serious events associated with taking the COVID-19 vaccines.

These financing mechanisms were crucial for accessing funds quickly and enabled AVATT to order and secure vaccine doses from credible vaccine manufacturers on behalf of African countries. It also reduced the influence of erratic and arbitrary external finance commitments on the timely implementation of the Africa Continental Vaccine Strategy. Beyond the financing mechanisms, the bi-monthly meetings between the ministries of health and finance ensured the effective coordination and mobilization of financing with distinct entities designated to manage the funds for transparency and accountability (Ngoy *et al.*, 2022).

Furthermore, the strong leadership of African institutions, private sector participation and support equally contributed to Africa’s vaccine financing efforts with donations from the MTN group, a leading African mobile network, and Mastercard Foundation.

Although the financing models used by African institutions are not necessarily new, the innovation here is an autonomous and African institution-led approach to coordinating and deploying external aid in a way that takes into account the continent’s peculiarities and needs. Some key takeaways that may be useful for financing other vaccine-preventable diseases in Africa include:

1. African governments are capable of investing in health, and, going forward, regional and national public health agencies will need more predictable sources of public funding to fund vaccines.
2. The partnership between the AU, Africa CDC, Afreximbank and AFDB should be consolidated to develop and implement innovative financing models for financing other vaccines.
3. AVAT and similar joint procurement mechanisms hold immense promise for the pooling, management and governance of both domestic and external aid, and should be preserved for future vaccine procurement to maximize resource efficiency (Ihekweazu, 2022).
4. High-level political engagement and committed leadership that prioritizes health can increase public funding and ultimately result in less reliance on external aid for vaccine financing.

Despite the WHO’s declaration of an end to COVID-19 as a global health emergency, Africa must continue to focus on its bold vision of vaccine self-reliance that will increase Africa’s manufacturing capacity from less than 1% to 60% by 2040. This bold vision calls on Africa’s leadership to take ownership by increasing public funding and sustaining the collaborative effort of African institutions in partnership with the private sector.

## Reflexivity Statement

My conviction as a lawyer and health policy professional is that equity ought to be the foundation of health laws, policies, programmes and interventions. Nonetheless, the present system of global health governance is deficient in robust accountability and enforcement measures in cases of default, particularly with regard to equity obligations. Thus, though equity is still a priority, I believe that countries should concentrate on boosting their domestic health investments to reduce the negative repercussions of depending too heavily on external aid, including donor exhaustion, conflicting priorities, lack of ownership and unsustainable interventions.

In the previous year, I conducted research on COVID-19 vaccine contracts, information access and their impact on vaccine equity. The interplay of politics, power and interests was glaringly apparent. Therefore, making the shift towards indigenous manufacturing of vaccines seems to be a prudent option from now on. Several countries have set their sights on producing vaccines domestically, but this remains a long-term vision. For now, they must concentrate on learning from financing models that give beneficiaries more decision-making power over vaccine procurement interventions. Successful models or models with good potential for success should be retained and replicated where adequate, while working toward pharmaceutical autonomy.
